# Assessing the relationship between the hospital grading system and medical resource utilization: evidence from China

**DOI:** 10.1186/s12962-025-00672-2

**Published:** 2026-01-05

**Authors:** Wanwen Jia, Xiaoli Wu

**Affiliations:** 1 China Research Institute of Regulation and Public Policy, Zhejiang University of Finance and Economics, No.18 Xueyuan Street, Hangzhou, Zhejiang 310018 China; 2 The Business School, Shaoxing University, No.1077 Chengnan Avenue, Shaoxing, Zhejiang 312000 China

**Keywords:** Hospital grading system, Medical resource utilization, Medical resource allocation, Primary healthcare

## Abstract

**Background:**

Improving medical resource utilization is a significant global challenge. In China, the hospital grading system serves as a key policy to promote collaboration and division of labor among institutions of different grades, with the goal of enhancing the overall performance. However, the medical resource utilization in China remains suboptimal. This study examines the relationship between the hospital grading system and medical resource utilization, offering insights for advancing the healthcare system.

**Methods:**

Using data from 31 provinces in China spanning 2010 to 2019, this study applies a dynamic panel data model to analyze the relationship between the hospital grading system and medical resource utilization. In addition, a mechanism analysis explores how the hospital grading system may influence utilization through the structure of resource allocation.

**Results:**

The hospital grading system demonstrates a significant negative association with the utilization of both outpatient and inpatient medical resources, with the impact varying across regions and levels of resource endowment. Furthermore, disparities in the allocation of resources between hospitals and primary healthcare institutions constitute a key mechanism through which the hospital grading system undermines overall utilization.

**Conclusions:**

This study finds that the hospital grading system is associated with a reduction in medical resource utilization in China. The results provide evidence to inform reforms aimed at revising hospital grading system standards, optimizing medical resource allocation and improving overall system performance in China, while also offering potential lessons for other countries facing similar healthcare challenges.

## Introduction

An effective healthcare system is a fundamental prerequisite for advancing the Healthy China initiative. To strengthen primary medical services and promote hierarchical diagnosis and treatment, China introduced the hospital grading management system, which integrates hierarchical management with hospital classification and emphasizes managed competition. This system represents a major policy implemented by the Chinese government to regulate healthcare quality [1]. It is important to note that the hospital grading system and the hierarchical diagnosis and treatment policy are distinct but closely related. The former provides a structural classification of institutions based on factors such as resources and service capacity, whereas the latter is a policy designed to guide patient flow and direct patients to the appropriate level of care.

The criteria for hospital grading cover five main dimensions: institutional scale, technical capacity, medical equipment, management level, and quality of care [2]. Healthcare institutions at different grades are designed to provide differentiated services and assume distinct responsibilities [3], with the goal of establishing a structured division of labor and functional complementarity across the system [4, 5]. Within this framework, China has developed a three-tier healthcare system comprising primary, secondary, and tertiary institutions [6]. Primary healthcare institutions were intended to provide basic healthcare services and act as gatekeepers in the medical system [7], yet this role has not been effectively realized in practice. Secondary institutions mainly deliver specialized care, while tertiary institutions focus on complex and critical cases [3].

However, evidence suggests that the hospital grading system has not fully achieved its intended objective of supporting hierarchical diagnosis and treatment. As a result, the quality and utilization of primary healthcare services in China remain suboptimal [8, 9]. While the hospital grading system was institutionally designed to establish a clear division of labor and promote collaboration across different levels of healthcare institutions, in practice its objectives have often deviated. Instead of fostering effective coordination, the system has been associated with stratified resource allocation, distorted healthcare-seeking behaviors, and ultimately lower utilization of healthcare resources.

As shown in Table 1, the proportion of healthcare technicians and hospital beds in primary healthcare institutions declined from 38.61% and 28.97% in 2010 to 33.16% and 20.24% in 2021, respectively. A similar downward trend is observed in service utilization: the share of outpatient visits and inpatient numbers decreased from 66.28% to 31.28%, while the proportion of inpatients fell from 31.28% to 15.72% over the same period. In contrast, these indicators have consistently increased in tertiary hospitals, where common and chronic diseases continue to absorb a large share of limited high-grade resources. Data from China’s Sixth National Health Services Statistical Survey Report further confirm this pattern, showing that the proportion of surveyed individuals seeking initial consultations at primary healthcare institutions, declining from 73.7% in 2008 to 67.5% in 2018. Furthermore, the inpatient ratio at primary healthcare institutions declined from 28.7% in 2008 to 17.8% in 2018, underscoring the persistent decline in patient reliance on primary healthcare services.

**Table 1:** The allocation and utilization of medical resources in China

Healthcare institutions	Medical resource allocation				Medical resource utilization			
	Proportion of healthcare technicians		Proportion of hospital beds		Proportion of outpatient visits		Proportion of inpatient numbers	
	2010	2021	2010	2021	2010	2021	2010	2021
Tertiary hospitals	23.50%	36.91%	25.88%	38.46%	13.96%	28.06%	24.53%	49.24%
Secondary hospitals	33.21%	24.94%	38.91%	32.66%	17.09%	15.77%	40.52%	30.15%
Primary hospitals	4.68%	4.99%	6.23%	8.64%	2.67%	2.72%	3.67%	4.90%
Primary healthcare institutions	38.61%	33.16%	28.97%	20.24%	66.28%	53.44%	31.28%	15.72%

Data Source: China Healthcare Statistical Yearbook. For the purposes of this study, the proportion calculations exclude specialized public health institutions and other institutions

The data in Table 1 further illustrates that China continues to face several challenges, including imbalanced allocation of healthcare resources [10], insufficient service capacity of primary healthcare institutions [2], and irrational patterns of medical service utilization [11]. The implementation of the hierarchical medical system has not achieved the intended outcomes [12].

Several studies suggest that the administrative, hierarchical model of resource allocation embedded in the hospital grading system is an important factor associated with the concentration of high-quality medical resources in high-grade hospitals, thereby squeezing the survival space of primary healthcare institutions [13]. This dynamic further exacerbates disparities in resource distribution across different levels of healthcare institutions [2]. As a result, inadequate resourcing in primary healthcare institutions constrains patient choices and contributes to the paradoxical coexistence of overcrowded high-grade hospitals with underutilized primary healthcare facilities. The unequal distribution and underutilization of medical resources thus remain central challenges in China’s healthcare sector [14, 15].

In response, the General Office of the State Council of China issued the “National Healthcare Service System Plan (2015–2020)” in 2015, which emphasized that some public hospitals in China had grown excessively large, while primary healthcare institutions lacked adequate service capacity. More recently, the 20th Party Congress report and the 2024 Government Work Report have underscored the need to accelerate the development of a hierarchical medical system and to promote the downward flow of high-quality healthcare resources.

In light of the aforementioned practical and policy context, exploring the causes of ineffective medical resources utilization and clarifying its underlying mechanisms is of substantial theoretical and practical significance. Such inquiry is crucial for strengthening primary healthcare service capacity, improving the allocation of medical resources, and advancing the construction of a hierarchical medical system. However, existing studies have predominantly focus on theoretical or qualitative analysis discussions, with limited empirical evidence regarding the relationship between the hospital grading system and medical resource utilization.

To address this gap, this study draws on data from China to conduct systematic theoretical and empirical research. Specifically, it seeks to answer the following questions: Does the hospital grading system adversely affect the utilization of medical resources? Are there heterogeneous effects? What are the potential pathways of influence? How can the allocation structure be optimized to enhance the utilization of medical resources?

The remainder of this study is structured as follows. Sect. “Literature review” reviews the relevant literature. Sect. “Theoretical analysis and research hypotheses” presents the theoretical framework and research hypotheses. Sect. “Methods” outlines the research design. Sect. “Results” discusses the empirical results. Section “Conclusions and discussion” concludes with policy implications, limitations, and suggestions for future research.

## Literature review

### Effects of the health grading system

Since the implementation of the hospital grading system, scholars have extensively examined its effects, particularly evaluating its policy implications from both supply- and demand-side perspectives.

From the supply-side perspective, healthcare institutions of different grades receive varying levels of resources and policy support, with hierarchical divisions creating disparities in the allocation of medical resources [16]. Within the administratively stratified model, higher-grade hospitals benefit from preferential government policies, greater fiscal investment, and access to advanced equipment, which may exacerbate polarization in resource allocation [13]. Consequently, high-quality medical resources tend to be concentrated in high-grade hospitals [17], while primary healthcare institutions face persistent shortages [18]. Importantly, resource allocation patterns shaped by the grading system on the supply side also influence patient choices on the demand side, thereby shaping overall medical resource utilization.

From the demand-side perspective, in a healthcare market characterized by information asymmetry, hospital grading system functions as a signal indicating medical service quality [3], shaping and constraining healthcare-seeking behavior [19, 20]. As hospital grade increases, its attractiveness to patients tends to rise. However, when patients rely on imperfect quality information, they may make suboptimal choices [21]. This dynamic leads many patients to favor high-grade hospitals for treatment [11, 22], even when their medical conditions do not necessitate tertiary-level resources [12]. Consequently, primary healthcare institutions experience persistently low demand, leaving them underutilized [23, 24]. Such patterns disrupt the intended three-tier healthcare system, hinder the implementation of the hierarchical medical system, and represent a persistent structural challenge in China’s healthcare sector [5].

Collectively, these studies suggest that the hospital grading system is strongly associated with the misallocation and underutilization utilization of medical resources in China. In sum, the evidence highlights significant shortcomings in the current hospital grading system and underscores the need for reform and improvement [13].

### Related studies on medical resource utilization

Existing research generally suggests that medical resource utilization in China remains suboptimal [25, 26], largely due to the weak capacity of the primary healthcare system, which has prompted numerous scholarly investigations into this issue. These studies focus on various aspects of medical resource utilization. Research in this area primarily examines efficiency measurement [27–29]and regional disparities [30–32]. The studies involve different types of medical institutions, such as rural township hospitals [33], community health centers [34, 35], primary-level maternal and child health hospitals [36], and the broader primary healthcare system [9]. Overall, existing evidence indicates that medical resource utilization in China remains relatively low [37], with inequitable and unfair utilization [38, 39].

Regarding the causes of inefficient medical resource utilization, Ye et al. (2021) [11]point out that the mismatch between patients’ conditions and doctors’ skills in the medical service market contributes to inefficient use of scarce medical resources. The overconcentration of patients and resources in high-grade hospitals and the underutilization in primary healthcare institutions jointly contribute to reduced system performance [40–42]. Furthermore, studies suggest that the greater the imbalance in resource allocation between high-grade hospitals and primary healthcare institutions, the lower the overall level of medical resource utilization [43].

### The health grading system and medical resource utilization

In recent years, research has increasingly examined the policy-related factors that influence medical resource utilization. For instance, Zhou et al. (2021) [2]found that hospitals often increase investments in equipment and medical personnel prior before grading assessments, which may contribute to inefficient use of resources. Similarly, studies have shown that the expansion of public hospitals has associated with resource waste [44]. Lv and Zhao (2018) [13] highlighted that the government-led hospital grading system plays a central role in shaping the uneven and irrational distribution of medical human resources between urban and rural areas. Sun et al. (2023) further argue that the comprehensive medical reform pilot strategy has had a positive effect on optimizing resource allocation [29].

Existing research provides valuable insights into the relationship between the hospital grading system and medical resource utilization in China. However, several gaps remain. First, although many studies focus on measuring efficiency or examining utilization levels, relatively few explore the institutional mechanisms through which hospital grading affects resource allocation, and a clear theoretical framework is still lacking. Second, the majority of existing work remains at the level of conceptual or theoretical discussion, while systematic empirical analysis of how the hospital grading system influences medical resource utilization is underdeveloped. Third, research on the specific pathways through which the hospital grading system shapes utilization remains limited and warrants further investigation.

Building on the above research gaps, this study makes three main contributions: (1) In terms of empirical analysis, it systematically examines the relationship between the hospital grading system on medical resource utilization and conducts heterogeneity analyses from the perspective of regional disparities and resource endowment, thus extending existing empirical work on these relationships. (2) From a theoretical perspective, it develops a framework to clarify the mechanisms through which the hospital grading system influences medical resource utilization via resource allocation, enriching theoretical discussions on the role of government regulatory policies in the healthcare sector. (3) From a policy perspective, it identifies the institutional drivers and mechanisms underlying inefficient utilization, providing empirical evidence and practical insights for optimizing resource allocation and improving the effectiveness of government regulation in China and other countries with comparable contexts.

## Theoretical analysis and research hypotheses

To explore the channels through which the hospital grading system affects the medical resource utilization, this study conducts a theoretical mechanism analysis from the perspective of resource allocation.

The initial aim of hospital grading management system was to establish a coordinated and complementary medical service system. In practice, however, the hospital grading system has deviated from its functional and service-based principles, evolving into a system largely determined by the quantity of medical resources. China’s administrative model strongly favors high-grade hospitals [3]. To obtain the benefits linked to higher grades, hospitals have expanded excessively [2], creating a “siphon effect” on primary healthcare resources that concentrates high-quality medical resources in high-grade hospitals while depriving primary healthcare institutions [13]. This imbalance undermines coordination mechanisms across institutions and generates irrational competition [12].

The persistent underdevelopment of primary healthcare is closely tied to the institutional incentives embedded in the grading system. Because the criteria emphasize quantitative indicators such as staff, beds, and equipment, investments are disproportionately directed toward higher-grade hospitals. Local governments lack incentives to fund primary institutions, while healthcare professionals prefer hospitals due to better pay and career prospects. These dynamics further weaken primary healthcare capacity and perpetuate systemic imbalances.

The structure of resource allocation also shapes patient behavior by signaling perceived quality [21, 45, 46]. Patients often select hospitals based on grade rather than medical need, with most favoring higher-grade institutions. This mismatch leaves primary care resources underutilized and contributes to overcrowding in tertiary hospitals [11]. Importantly, bypassing primary care reflects not cultural preference but a rational response to institutional shortcomings, such as weak referral mechanisms and inadequate primary capacity. These deficiencies reduce patient trust, exacerbate concentration in higher-grade hospitals, and intensify inefficiencies. Moreover, overlapping service provision across different levels of institutions further amplifies resource misallocation and inefficiency.

**Fig. 1:**
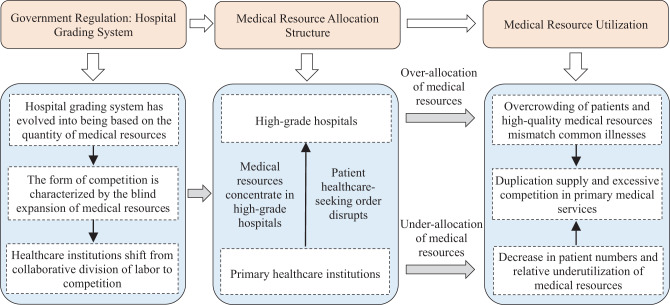
Influence mechanism

In summary, this study argues that the vertical structure of medical resource allocation is a key mechanism through which the hospital grading system influences the medical resource utilization, as illustrated in Fig. 1. Based on the above analysis, the following hypotheses are proposed:

### Hypothesis 1:

The hospital grading system is associated with lower levels of medical resource utilization.

### Hypothesis 2:

The hospital grading system may contribute to reduced medical resource utilization by exacerbating disparities in medical resource allocation.

## Methods

### Model setting

#### Dynamic panel model

Previous studies suggest that the utilization of medical resources exhibits temporal persistence, whereby current utilization levels may be influenced by those of the preceding period (Zheng and Shen, 2019) [47]. To capture this dynamic feature, this study incorporates a one-period lag of the dependent variable in the regression equation. Accordingly, a dynamic panel model is employed to estimate the relationship between the hospital grading system and medical resource utilization. This approach also helps address potential endogeneity and unobserved heterogeneity, which are common challenges in health system research. The econometric model is specified as follows:

1 $${y_{it}} = {\partial _0} + {\partial _1}L.{y_{it}} + {\partial _2}H{G_{it}} + {\partial _c}Contro{l_{it}} + {\mu _i} + {\varepsilon _{it}}$$

where ${y_{it}}$ represents the level of outpatient medical resource utilization $OU{E_{it}}$ and inpatient medical resource utilization $IU{E_{it}}$ of province $i$ in period $t$.$L.{{\rm{y}}_{it}}$denotes the one-period lag of ${y_{it}}$. $H{G_{it}}$ stands for the level of hospital grading system in province $i$ in period $t$. $Contro{l_{it}}$ denotes the set of control variables affecting $OUE$ and $IUE$. ${\mu _i}$ represents province fixed effects, and ${\varepsilon _{it}}$ is the random error term.

#### Analysis of mechanisms

Building on the theoretical framework, this study further investigates whether the structure of medical resource allocation mediates the relationship between the hospital grading system and medical resource utilization. The econometric specification is as follows:

2 $$MA{S_{it}} = {\beta _0} + {\beta _1}L.MA{S_{it}} + {\beta _2}H{G_{it}} + {\beta _c}Contro{l_{it}} + {\mu _i} + {\varepsilon _{it}}$$

3 $${y_{it}} = {\sigma _0} + {\sigma _1}L.{y_{it}} + {\sigma _2}H{G_{it}} + {\sigma _3}MA{S_{it}} + {\sigma _c}Contro{l_{it}} + {\mu _i} + {\varepsilon _{it}}$$

where $MA{S_{it}}$ represents the medical resource allocation structure of province $i$ in period $t$. $L.MA{S_{it}}$ denotes its one-period lag. The remaining variables are defined as in Eq. 1. The testing procedure is as follows. First, if the regression coefficient of HG on OUE/IUE in the baseline model is statistically significant, two additional regressions are estimated: (2) the regression of HG to MAS, and (3) the regression of HG and MAS jointly on OUE/IUE. Finally, the significance and magnitude of the estimated coefficients ${\beta _2}$, ${\sigma _2}$, and ${\sigma _3}$ are used to assess whether MAS mediates the relationship between HG and OUE/IUE.

### Variable selection and data description

#### Variable selection

Explained variable. The explained variable in this study is medical resource utilization, which include both outpatient and inpatient services. This study focuses on utilization levels, measured by the volume of services provided, as they directly reflect the hospital grading system’s influence on patient flows and resource allocation. Following Zheng and Shen (2019) [47], outpatient medical resource utilization (*OUE*) is measured by the average number of outpatient visits per resident at all medical institutions, and inpatient medical resource utilization (*IUE*) is measured by the natural logarithm of the annual hospitalization rate. These indicators capture the extent of service provision, rather than efficiency in the traditional sense of maximizing outputs given inputs or minimizing inputs for given outputs.

Core explanatory variables. The hospital grading system (*HG*) is defined as an institutional classification scheme that categorizes healthcare institutions into three levels—primary, secondary, and tertiary—according to the official standards established by the Chinese government. The proportion of tertiary hospitals in a region serves as a direct, quantifiable measure and a strong indicator of the policy’s implementation intensity. To operationalize this, the study follows Lv and Zhao (2018) [13] and uses the proportion of tertiary hospitals to the total number of hospitals in each province-year. This indicator captures both regional differences in hospital distribution across provinces and temporal evolution of the grading structure as the reform progressed from 2010 to 2019.

Mechanism variable. Based on the theoretical analysis, the mechanism variable in this study is defined as the medical resource allocation structure (*MAS*). Healthcare resource allocation refers to the distribution of resources across various sectors, regions, and levels of institutions, with the objective of optimizing their utilization. The ratio of resource allocation across different tiers and categories of medical institutions serves as a key indicator for assessing the rationality of the medical resource allocation structure. This study specifically focuses on disparities in resource distribution between hospitals and primary healthcare institutions. Following Zou (2014) [48], the coefficient of variation method is used to measure disparities in allocation. To enhance the robustness, the Theil Index method is also employed as an alternative measure of structural disparities in medical resource allocation.

(1) Coefficient of Variation Method. Medical resources primarily consist of human and material resources. Accordingly, this study calculates the medical human resources allocation structure (*MAS_H*) and medical material resources allocation structure (*MAS_M*) separately. Human resources are measured by the number of healthcare technicians, while to capture internal structural differences, the number of practicing (assistant) physicians and registered nurses are also included as supplementary indicators. Material resources are represented by the number of hospital beds, which reflect both the hardware capacity and the development scale of medical institutions. The coefficient of variation (CV) is computed using the following formula:

4 $$cv = {{\sqrt {\sum\limits_{i = 1}^n {{{({x_i} - \overline x )}^2}/n} } } \over {\overline x }}$$

A smaller $cv$ value, indicates a lower vertical disparity in medical resource allocation, while a larger CV value reflects a higher degree of disparity. Here, $i$ represents the group of hospitals or primary healthcare institutions, ${x_i}$ denotes the specific medical resource indicator for each group, and $\overline x = \sum\nolimits_{i = 1}^n {{x_i}} /n$, refers to the mean of the resource indicator across groups.

The entropy method determines weights based on variations in the indexes. This study applies this method to assign weights to the allocation structures of medical human and material resources. The steps of the procedure are as follows:

First, various index data are normalized using the following formula:

5 $${Y_{{\rm{ij}}}} = {{{X_{ij}} - \min \left( {{X_{ij}}} \right)} \over {\max \left( {{X_{ij}}} \right) - \min \left( {{X_{ij}}} \right)}}$$

Here, ${Y_{{\rm{ij}}}}$ represents the normalized index, $i$ denotes the province, and $j$ represents the human and material resource indicators used to measure the structure of medical resource allocation structure, $j = 1,{\rm{ }}2, \ldots ,K$, with $K$ representing the total number of indexes.

Second, the information entropy for each index is calculated using the following formulas:

6 $${P_{{\rm{ij}}}} = {{{Y_{ij}}} \mathord{\left/ {\vphantom {{{Y_{ij}}} {\sum\limits_{i = 1}^n {{Y_{ij}}} }}} \right. \kern-\nulldelimiterspace} {\sum\limits_{i = 1}^n {{Y_{ij}}} }}$$

7 $${E_j} = - {1 \over {\ln ({\rm{n)}}}} \times \mathop \sum \limits_{i = 1}^n {P_{ij}}\ln {P_{ij}}$$

Third, weights are determined based on the following formula:

8 $${W_j} = {{1 - {E_j}} \over {K - \sum {{E_j}} }}$$

Using the method described above, the weights of medical human and material resources were calculated for each year, yielding average values of 0.4314 and 0.5686 for the period from 2010 to 2019, respectively. These weights were then used to calculate the structure of medical resource allocation for each year.

(2) Theil Index Method. To mitigate measurement errors associated with a single indicator, this study also employs the Theil index to assess the structure of medical resource allocation. The Theil index, initially introduced by Theil through the concept of total entropy from information theory, was originally used to measure income disparities across individuals or regions. Over time, this method has become an important tool for evaluating the structure of medical resource allocation. The Theil index reflects the magnitude of differences between individuals and the mean, with a higher value indicates a greater disparity. As referenced in the studies by Liu et al. (2013) [49] and Zhu et al. (2024) [50], the calculation formula for the Theil index is as follows:

9 $$T = {1 \over n}\sum\limits_{i = 1}^n {{{{x_i}} \over {\overline x }}\log ({{{x_i}} \over {\overline x }})} $$

Where, $i$ represents the hospital or primary healthcare institution group, ${x_i}$ represents various medical resource indicators for hospitals or primary healthcare institutions, $\overline x = \sum\nolimits_{i = 1}^n {{x_i}} /n$, denotes the mean values of these medical resource indicators. Based on the Theil index method, this study calculates the medical human resource structure and medical material resource structure across different provinces of China from 2010 to 2019. Weights are assigned using the entropy method, and the overall structure of medical resource allocation (*TMAS*) is subsequently computed based on these weights.

Control variables. To account for various factors that might influence the outpatient and inpatient medical resource utilization, and drawing on the existing literature [5, 13, 51], this study selects the following variables as control variables:

1. Intensity of fiscal health expenditure (*fhexp*). Fiscal health expenditure and allocation are critical factors influencing the allocation and utilization of medical resources. Therefore, this study uses the proportion of local fiscal health expenditure to the local general budget expenditure as a proxy for fiscal health expenditure intensity.
2. Urbanization rate (*urban*). The urbanization rate reflets the economic development and healthcare level of a province and is closely related to the medical resources utilization. This study measures the urbanization rate as the percentage of the urban population at the end of the year relative to the total population in the region.
3. Economic development level (*pgdp*). Economic development level significantly impacts the supply capacity of medical services. Following the approach of most studies, it is measured by the natural logarithm of the region’s per capita Gross Domestic Product (GDP). To account for inflation, this study adjusts per capita GDP to the 2010 base year using the regional per capita GDP index.
4. Population aging (*aging*). As the population ages, the demand for medical services rises, thereby affecting the utilization of medical resources. This study uses the percentage of the population aged 65 and above relative to the total population as the measure for population aging.
5. Population density (*den*). The population density of a province has a direct impact on the utilization of medical resources. This study uses the natural logarithm of the number of permanent residents per square kilometer at the end of the year as a proxy for population density.
6. Educational attainment (*edu*). The level of education affects patients’ choices of medical institutions, which in turn impacts medical resource utilization. This study represents educational attainment as the percentage of the population aged 6 and above with a junior college degree or higher relative to the total population.
7. Health insurance coverage rate (*hicr*). Health insurance reduces the price elasticity of patient demand and subsequently influences medical resource utilization. Following the research by Song et al. (2019) [52], this study uses the urban employee health insurance coverage rate as the measurement index, calculated as the ratio of participants in urban employee basic health insurance to the total population in the region.

#### Data source

The data used in this research were sourced from several official publications: the China Healthcare Statistical Yearbook, China Healthcare Development Statistical Bulletin, China Statistical Yearbook, China Population & Employment Statistical Yearbook, and China Labor Statistical Yearbook. The sample period spans from 2010 to 2019, selected for two primary reasons: First, due to the availability of key indicators, which have only been publicly accessible since 2010. Second, this timeframe ensures the stability and reliability of the results, as the COVID-19 outbreak in China significantly impacted some indicators, introducing substantial variability. Consequently, data collection is restricted to the end of 2019.

In this study, the analysis is conducted within the framework of China’s hospital grading system. The dataset includes both public and private hospitals; however, it should be noted that high-level hospitals, particularly large tertiary hospitals, are predominantly public. As such, the disparities discussed primarily reflect the structural imbalance between large public hospitals and primary healthcare institutions.

Data on healthcare human resources in primary healthcare institutions are missing for the years 2010 and 2011. Based on the classification of primary healthcare institutions in China, this study estimates the missing values as follows: human resources in primary healthcare institutions = total personnel in healthcare institutions - (personnel in hospitals + personnel in maternal and child health hospitals (institutes, stations) + personnel in disease control centers + personnel in health supervision offices (centers))^1^**.** Using the published data from 2012 to 2019, the overestimation ranged between 1.004 and 1.141. These overestimated portions of the 2010 and 2011 data were adjusted to improve result accuracy.

After supplementing the missing data using the above method, and considering that panel data can mitigate multicollinearity issues among variables, this study focuses on the 31 provinces within mainland of China, excluding Hong Kong, Macau, and Taiwan. Logarithmic transformations were applied to certain variables to correct for heteroskedasticity. Definitions of variables and descriptive statistics are provided in Table 2, which offers a summary of the data and helps identify potential outliers or issues that may need to be addressed in further analysis.

**Table 2:** Variable definitions and descriptive statistics

Variable	Definition	Mean	Min	Max	SD	N
OUE	Total number of medical visits/resident population	5.314	2.310	11.650	1.874	310
IUE	The natural logarithm of the annual hospitalization rate (number of hospitalizations/resident population)	2.645	0.531	3.182	0.300	310
HG	Number of Tertiary hospitals/total number of hospitals	0.077	0.019	0.158	0.031	310
MAS	Medical resource allocation structure (Coefficient of Variation)	0.495	0.218	0.756	0.113	310
TMAS	Medical resource allocation structure (Theil Index)	0.063	0.011	0.158	0.031	310
fhexp	Local fiscal health expenditure/local general budget expenditure	0.074	0.040	0.106	0.015	310
urban	The urban population at the end of the year/total population	0.561	0.227	0.896	0.134	310
pgdp	The natural logarithm of the region’s per capita GDP	10.642	9.464	11.905	0.476	310
aging	Individuals aged 65 and above relative/total population	0.099	0.048	0.163	0.023	310
den	The natural logarithm of the number of permanent residents per square kilometer at the end of the year	8.982	7.180	10.213	0.645	310
edu	Population aged 6 and above with a junior college degree or higher/total population	0.132	0.024	0.505	0.072	310
hicr	Number of urban workers with basic health insurance/total population	0.217	0.078	0.781	0.135	310

## Results

### Baseline regression analysis

For dynamic panel models, commonly used estimation methods include Difference GMM and System GMM. System GMM makes use of more information than Difference GMM, resulting in higher estimation efficiency. Therefore, this study primarily employs the one-step System GMM estimation method, while addressing heteroscedasticity issues through robust standard error corrections. The serial correlation of the disturbance term is evaluated using the p-values from the AR (1) and AR (2) tests. When the number of instrumental variables exceeds the number of endogenous variables, an over-identification test is necessary for the instrumental variables. Arellano and Bond (1991) [53] noted that, in the presence of heteroscedasticity in the disturbance terms, the Sargan test tends to over-reject the null hypothesis. As a result, they recommended performing the Sargan test after two-stage estimation. Therefore, the Sargan test p-values reported in this study are derived from two-stage estimation procedure.

This study examines the impact of the hospital grading system on medical resource utilization, with the empirical results presented in Table 3. Columns (1) and (2) of Table 3 display the regression results for outpatient utilization, while columns (3) and (4) of Table 3 present those for inpatient utilization. The regression analysis reveals that the coefficient of the core explanatory variable (*HG*) is significantly negative. These findings provide evidence of a statistically significant negative relationship between the hospital grading system and both outpatient and inpatient medical resource utilization. Thus, Hypothesis 1 is supported.

**Table 3:** Results of baseline regression

Variables	OUE		IUE	
	(1)	(2)	(3)	(4)
L. OUE	1.021***	0.784***		
	(0.022)	(0.078)		
L. IUE			0.878***	0.678***
			(0.024)	(0.036)
HG	−3.214**	−5.675**	−0.770**	−2.029***
	(1.567)	(2.507)	(0.348)	(0.551)
fhexp		−2.953		0.520
		(3.293)		(0.675)
urban		−1.023		−0.274
		(0.830)		(0.424)
pgdp		0.331		0.165
		(0.302)		(0.111)
aging		1.605		1.408*
		(2.288)		(0.722)
den		0.099		−0.016
		(0.096)		(0.035)
edu		−2.585**		−0.278
		(1.106)		(0.202)
hicr		6.258***		0.364
		(1.101)		(0.268)
Constant	0.326***	−3.052	0.440***	−0.620
	(0.107)	(2.672)	(0.077)	(0.891)
AR (1)	0.021	0.026	0.007	0.005
AR (2)	0.563	0.475	0.796	0.323
Sargan	0.978	0.996	0.985	0.996
Sample size	279	279	279	279

Note: L. denotes the one-period lag of the variable. * denotes *p* < 0.1, ** denotes *p* < 0.05, *** denotes *p* < 0.01, robust standard errors are reported in parentheses. AR (1) and AR (2) represent the p-values for the serial correlation tests, Sargan indicates the p-value for the over-identification test. The symbols in the following table are the same as in this table

The observed negative association between the grading system and medical resource utilization can be attributed to the current healthcare landscape in China. Specifically, some large hospitals, aiming to enhance their status by signaling higher medical quality through grading, have overexpanded their scale, leading to the concentration of resources in high-grade institutions. The misallocation of high-quality medical resources to primary healthcare services is associated with a decrease overall utilization. Meanwhile, primary healthcare institutions, with lack access to these resources, struggle to provide adequate services, resulting in underutilized resources. Furthermore, the overlap in service provision across different grades of healthcare institutions fosters irrational competition in the primary healthcare market. This disordered competition disrupts patient care-seeking behavior, creating an imbalance between supply and demand, ultimately decreases the medical resource utilization.

In this study, certain control variables, including the intensity of fiscal health expenditure, urbanization rate, economic development level, and population density, do not demonstrate significant effects on medical resource utilization. However, the coefficient of population aging on inpatient medical resource utilization is significantly positive, indicating that an aging population increases the annual hospitalization rate. In contrast, the coefficient of educational attainment on outpatient medical resource utilization is significantly negative, suggesting that higher education levels are associated with a decrease in the average number of outpatient visits. Additionally, the coefficient of the health insurance coverage rate on outpatient medical resource utilization is significantly positive, indicating that increased medical insurance coverage reduces the price elasticity of demand for outpatient services, thereby enhancing medical resource utilization.

### Robustness test

#### Replacement of empirical estimate method

In addition to one-step system GMM, one-step difference GMM is also commonly used for estimating dynamic panel models. This study conducts an initial robustness test by substituting the estimation method with one-step difference GMM. The empirical results are presented in columns (1) and (2) of Table 4. The results show that the coefficient of the hospital grading system remains significantly negative, indicating that the findings are robust to alternative empirical estimation methods.

**Table 4:** Results of the robustness test

Variables	One-step differential GMM		Replacing the explanatory variable		Increasing control variables	
	(1)	(2)	(3)	(4)	(5)	(6)
	OUE	IUE	OUE	IUE	OUE	IUE
L. OUE	0.629***		0.963***		0.797***	
	(0.112)		(0.043)		(0.078)	
L. IUE		0.505***		0.681***		0.662***
		(0.085)		(0.041)		(0.042)
HG	−4.359*	−1.689***			−5.067**	−1.779***
	(2.424)	(0.506)			(2.541)	(0.546)
THG			−5.683*	−1.819*		
			(3.037)	(1.064)		
fhexp	−3.398	−0.532	−3.835**	0.809	−1.866	0.450
	(2.391)	(0.649)	(1.955)	(0.494)	(2.692)	(0.570)
urban	−0.026	−0.135	−0.061	−0.101	−0.956	−0.263
	(1.401)	(0.478)	(0.779)	(0.396)	(0.760)	(0.396)
pgdp	0.600	0.380***	0.181	0.092	0.292	0.083
	(0.377)	(0.136)	(0.213)	(0.073)	(0.365)	(0.094)
aging	−1.749	−0.225	−1.311	1.566**	1.334	1.246*
	(1.530)	(0.325)	(1.797)	(0.767)	(2.241)	(0.640)
den	0.040	0.027	0.076	−0.012	0.109	−0.006
	(0.050)	(0.026)	(0.071)	(0.029)	(0.081)	(0.030)
edu	−1.994**	−0.098	−2.648***	−0.359*	−2.794**	−0.170
	(0.963)	(0.109)	(0.595)	(0.214)	(1.151)	(0.165)
hicr	7.240***	0.432	2.363***	0.146	5.807***	0.541**
	(1.375)	(0.309)	(0.830)	(0.254)	(1.143)	(0.247)
doct					0.120***	−0.019
					(0.045)	(0.014)
bed					−0.036	0.027**
					(0.042)	(0.012)
Constant	−5.176*	−2.764***	−1.679	−0.038	−2.908	0.047
	(2.934)	(0.992)	(2.042)	(0.602)	(3.229)	(0.784)
AR (1)	0.008	0.058	0.014	0.002	0.014	0.009
AR (2)	0.301	0.407	0.165	0.232	0.867	0.228
Sargan	0.806	0.749	1.000	0.997	0.997	0.995
Sample size	248	248	279	279	279	279

#### Substitution of explanatory variables

Grade A tertiary hospitals exert a significant “siphon effect” on primary healthcare resources, and their proportion relative to the total number of hospitals is used as an indicator of the hospital grading system. This study replaces the core explanatory variable with the proportion of Grade A tertiary hospitals (*THG*) to assess the robustness of the baseline regression model. The regression results are presented in columns (3) and (4) of Table 4. The findings indicate that the hospital grading system continues to exert a significant negative impact on both outpatient and inpatient medical resource utilization, with estimated coefficients of −5.683 and −1.819 respectively. This demonstrates that even after substituting the measurement indicators of the core explanatory variable, the baseline regression results remain valid.

#### Incorporating control variables

To further address the issue of potential omitted variables that may affect the results, this study incorporates two additional control variables in the analysis: the number of practicing (assistant) physicians per thousand population (*doct*) and the number of beds in medical and health institutions per thousand population (*bed*), following the approach advocated by Zhou and Li (2023) [54]. The results of this extended model are presented in columns (5) and (6) of Table 4, showing that the coefficient of the hospital grading system remains significantly negative. These findings are consistent with those of the baseline regression, indicating that the results remain robust even after controlling for additional variables.

### Heterogeneity analysis

The levels of economic development, overall medical resource allocation, and the structure of these resources vary significantly across regions in China. To better understand the impact of the hospital grading system on outpatient and inpatient medical resource utilization, this study conducts a heterogeneity analysis, considering both regional differences and medical resource endowment.

#### Regional heterogeneity

To explore whether regional differences affect the impact of the hospital grading system on medical resource utilization, this study divides China into two geographic regions (*Central-Western* and *Eastern*), based on the regional classification standards outlined in the China Healthcare Statistical Yearbook. The results are presented in Table 5.

**Table 5:** Results of regional heterogeneity

Variables	OUE		IUE	
	(1)	(2)	(3)	(4)
	Central-Western	Eastern	Central-Western	Eastern
L. OUE	1.007***	0.808***		
	(0.085)	(0.114)		
L. IUE			0.660***	0.809***
			(0.043)	(0.092)
HG	−4.854**	−8.449***	−1.423*	−0.050
	(2.245)	(2.232)	(0.798)	(0.407)
fhexp	−5.050*	−12.329*	−0.882	1.116**
	(2.979)	(7.113)	(0.960)	(0.511)
urban	−1.143	−5.328***	0.539	−0.620***
	(0.966)	(1.418)	(0.401)	(0.178)
pgdp	−0.109	2.069**	0.065	0.091
	(0.187)	(0.815)	(0.045)	(0.073)
aging	4.848*	−11.448***	2.121***	0.324
	(2.722)	(3.026)	(0.618)	(0.347)
den	0.017	0.000	−0.008	−0.024
	(0.055)	(0.213)	(0.026)	(0.029)
edu	−1.615*	−1.835	−0.482*	−0.191**
	(0.846)	(1.240)	(0.250)	(0.080)
hicr	5.478***	3.694***	0.167	0.365**
	(1.838)	(1.218)	(0.712)	(0.159)
Constant	1.256	−15.738**	0.078	−0.047
	(1.845)	(6.715)	(0.456)	(0.543)
AR (1)	0.006	0.094	0.009	0.036
AR (2)	0.506	0.815	0.334	0.326
Sargan	0.974	1.000	1.000	1.000
Sample size	180	99	180	99

The results in columns (1) and (2) of Table 5 indicate a significant negative association between the hospital grading system and outpatient utilization in both the central-western and eastern regions, suggesting broadly consistent effects across regions. This consistency supports the robustness of the baseline findings regarding outpatient utilization. For inpatient utilization, however, the regression results in columns (3) and (4) of Table 5 reveal that the hospital grading system has a significant negative impact in the central-western region, while the coefficient in the eastern region remains negative but statistically insignificant.

One possible explanation is that the eastern region benefits from relatively higher levels of economic development and medical service capacity. In contrast, the central-western region suffers from more limited healthcare resources, with primary healthcare institutions often unable to adequately meet patients’ medical needs. As tertiary hospitals are widely perceived to represent higher medical quality, patients’ tendency to gravitate toward them becomes particularly pronounced. Consequently, the hospital grading system appears to exert a stronger adverse influence on inpatient medical resource utilization in such region. The results further suggest that, compared with outpatient services, patients hold higher quality expectations for inpatient care, and the impact of the hospital grading system on inpatient medical resource utilization appears to differ across regions.

In conclusion, these findings highlight the need to carefully consider regional disparities when assessing the impact of the hospital grading system on medical resource utilization and when designing healthcare reform policies aimed at improving system-wide performance.

#### Medical resource endowment heterogeneity

Practicing (assistant) physicians are a crucial component of medical human resources and play a significant role in influencing patients’ choices for medical treatment. This study uses the number of practicing (assistant) physicians per thousand population as the primary measurement indicator. Based on the average number of practicing (assistant) physicians per thousand population in 2019 across 31 provinces, the provinces are categorized the into two groups: high medical resource endowment (*High-mre*) and low medical resource endowment (*Low-mre*) regions. The estimation results are presented in Table 6.

**Table 6:** Results of medical resource endowment heterogeneity

Variables	OUE		IUE	
	(1)	(2)	(3)	(4)
	High-mre	Low-mre	High-mre	Low-mre
L. OUE	0.933***	1.015***		
	(0.083)	(0.079)		
L. IUE			0.827***	0.731***
			(0.061)	(0.046)
HG	−7.577***	−2.432	−0.248	−1.543*
	(2.927)	(2.396)	(0.626)	(0.885)
fhexp	−5.961	−6.724**	0.676	−0.887
	(5.802)	(2.836)	(0.677)	(1.207)
urban	−1.516	−0.193	−0.774***	0.500
	(1.141)	(1.230)	(0.155)	(0.355)
pgdp	0.462	−0.141	0.205***	−0.008
	(0.516)	(0.168)	(0.065)	(0.049)
aging	−4.338**	4.170**	−0.493	2.724***
	(1.686)	(1.934)	(0.424)	(0.633)
den	0.186	−0.026	−0.039**	−0.032
	(0.137)	(0.086)	(0.017)	(0.036)
edu	−1.958*	−1.616*	−0.120	−0.291
	(1.069)	(0.904)	(0.133)	(0.275)
hicr	4.306***	2.368	0.094	−0.047
	(1.163)	(1.559)	(0.138)	(0.434)
Constant	−4.479	2.004	−0.884	0.870
	(4.769)	(1.897)	(0.578)	(0.721)
AR (1)	0.061	0.013	0.011	0.073
AR (2)	0.692	0.549	0.645	0.141
Sargan	0.999	0.999	0.998	1.000
Sample size	135	144	135	144

The results in columns (1) and (2) of Table 6 indicate that the hospital grading system is significantly associated with lower outpatient medical resource utilization in high medical resource endowment regions, whereas the coefficient remains negative but not statistically significant in low endowment regions. This may be explained by the fact that high-grade hospitals and high-quality medical resources are concentrated in high-resource regions, leading to significant disparity in outpatient medical resource allocation across healthcare institutions grades. As a result, outpatient departments in large hospitals tend to be overcrowded, with high-quality medical resources allocated excessively to common diseases, thereby reducing actual outpatient resource utilization. These findings suggest that the hospital grading system’s adverse impact on outpatient utilization is more pronounced in high-resource regions.

Columns (3) and (4) of Table 6 reveal that the coefficient of the hospital grading system for inpatient medical resource utilization is not statistically significant in high-resource regions but is significantly negative in low-resource regions. One possible explanation is that limited medical resources in low-resource regions encourage a stronger patient preference for inpatient services at high-grade hospitals, resulting in pronounced disparities in treatment choices. Consequently, inpatient resources in primary healthcare institutions are underutilized, leading to low overall inpatient utilization in these regions.

### Mechanism analysis

To examine whether the structure of medical resource allocation influences the relationship between the hospital grading system and medical resource utilization, this study conducts the following analysis.

#### Analysis of mechanism variables

**Fig. 2:**
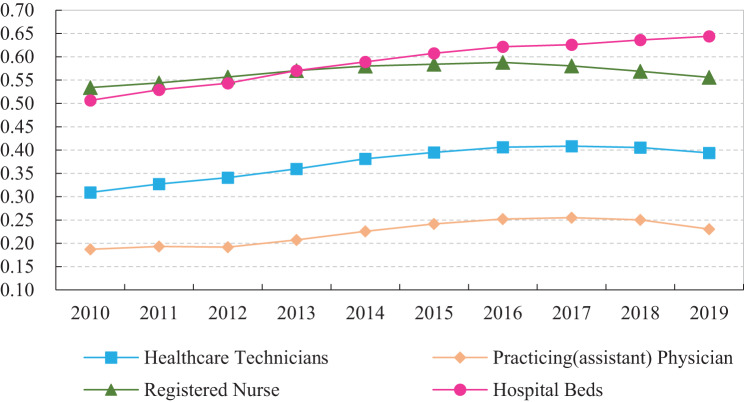
Medical resource allocation structure in China (2010–2019)

Following Eq. 2 and Eq. 3, this study calculates the allocation structure of different types of medical resources across China’s 31 provinces from 2010 to 2019. To assess the overall evolutionary trends, the average values of the medical resource allocation structure are computed, as shown in Fig. 2.

Figure 2 illustrates key characteristics of the medical resource allocation structure between hospitals and primary healthcare institutions in China from 2010 to 2019:

1. Expanding disparity in the allocation of medical human resources. From 2010 to 2019, the Coefficient of variation for healthcare technicians increased from 0.309 to 0.394, for practicing (assistant) physicians from 0.187 to 0.230, and for registered nurses from 0.534 to 0.556. These findings indicate that, in recent years, the disparity in the medical human resource allocation structure between hospitals and primary healthcare institutions has steadily widened. The disparity is more pronounced for registered nurses than for practicing (assistant) physicians.
2. Deficit of medical material resources in primary healthcare institutions. From 2010 to 2019, the Coefficient of variation for the number of hospital beds increased from 0.507 to 0.644, signifying a growing disparity in bed capacity between hospitals and primary healthcare institutions. Within the administratively hierarchical healthcare system, social resources are concentrated in high-grade hospitals, diminishing the competitiveness of primary healthcare institutions. The shortage of medical resources significantly constrains the service capacity of primary healthcare institutions.

#### Empirical results of influence mechanisms

To further explore whether the hospital grading system exerts an indirect effect on medical resource utilization through medical resource allocation (MAS), this study employs the mediation model specified in Eq. 2 and Eq. 3. The corresponding regression results are summarized in Table 7.

**Table 7:** Results of the mechanism test for MAS (measured by coefficient of variation)

Variables	(1)	(2)	(3)	(4)	(5)
	OUE	MAS	OUE	IUE	IUE
L.OUE	0.784^***^		0.857^***^		
	(0.078)		(0.071)		
L.MAS		0.894^***^			
		(0.062)			
L.IUE				0.678^***^	0.729^***^
				(0.036)	(0.030)
HG	−5.675^**^	0.325^*^	−5.105^**^	−2.029^***^	−1.408^**^
	(2.507)	(0.166)	(2.310)	(0.551)	(0.660)
MAS			−2.061^**^		−0.380^**^
			(1.043)		(0.156)
fhexp	−2.953	0.380	−2.427	0.520	1.434^*^
	(3.293)	(0.236)	(2.747)	(0.675)	(0.800)
urban	−1.023	0.056	−0.064	−0.274	−0.155
	(0.830)	(0.048)	(0.852)	(0.424)	(0.301)
pgdp	0.331	−0.035^***^	0.506^*^	0.165	0.112
	(0.302)	(0.011)	(0.297)	(0.111)	(0.076)
aging	1.605	−0.163	−0.435	1.408^*^	1.616^***^
	(2.288)	(0.106)	(1.660)	(0.722)	(0.490)
den	0.099	−0.018	−0.014	−0.016	−0.041
	(0.096)	(0.013)	(0.081)	(0.035)	(0.026)
edu	−2.585^**^	−0.004	−1.669^*^	−0.278	−0.058
	(1.106)	(0.042)	(0.923)	(0.202)	(0.188)
hicr	6.258^***^	−0.033	4.041^***^	0.364	0.089
	(1.101)	(0.061)	(0.956)	(0.268)	(0.218)
Constant	−3.052	0.540^***^	−3.311	−0.620	0.044
	(2.672)	(0.151)	(2.645)	(0.891)	(0.628)
AR (1)	0.026	0.016	0.014	0.005	0.003
AR (2)	0.475	0.249	0.345	0.323	0.271
Sargan	0.996	0.977	0.990	0.996	1.000
Sample size	279	279	279	279	279

The regression results in column (2) of Table 7 demonstrate that the coefficient of HG on MAS is significant at the 1% level, indicating that the hospital grading system exacerbates the disparity in medical resource allocation between hospitals and primary healthcare institutions. The results in column (3) of Table 7 present the regression results after including the mediation variable MAS. The coefficient of HG on OUE is significantly negative (−5.105), which represents a reduction in magnitude compared to the value of −5.675 in column (1) of Table 7. This suggests that the hospital grading system is associated with a reduction in outpatient medical resource utilization by exacerbating the disparity in medical resource allocation. Similarly, the regression results in columns (1), (4) and (5) of Table 7 reveal that the hospital grading system is linked to lower inpatient medical resource utilization via the medical resource allocation structure. Thus, Hypothesis 2 is supported.

The hospital grading system appears to be associated with a reduction in medical resource utilization by widening the disparity in medical material resource allocation. The mechanisms underlying this are illustrated in Fig. 1: First, the administratively hierarchical hospital grading system exacerbates the initial allocation disparity of medical resources across different grades of medical institutions. Second, there is an imbalance in the allocation, with insufficient resources in primary healthcare institutions and excessive allocation in high-grade healthcare institutions. Lastly, this allocation influences patients’ treatment choices, fostering a stronger preference for high-grade hospitals. As a result, the medical resource utilization of both primary healthcare institutions and high-grade hospitals is affected, ultimately leading to a decline in the utilization of the entire medical system.

To test the robustness of the aforementioned mechanism analysis, this study separately uses the unweighted medical human resource allocation structure (*MAS_H*) and the unweighted medical material resource allocation structure (*MAS_M*) as mediating variables for the mechanism analysis. The regression results are presented in Tables 8 and 9, respectively.

**Table 8:** Results of the mechanism test for MAS_H (measured by coefficient of variation)

Variables	(1)	(2)	(3)
	MAS_H	OUE	IUE
L.OUE		0.926^***^	
		(0.058)	
L.MAS_H	0.894^***^		
	(0.102)		
L.IUE			0.746^***^
			(0.032)
HG	0.391^*^	−4.978^***^	−1.118^*^
	(0.224)	(1.604)	(0.599)
MAS_H		−1.382^**^	−0.227^*^
		(0.590)	(0.125)
fhexp	0.983^**^	1.641	1.295
	(0.444)	(2.382)	(0.923)
urban	−0.050	0.174	−0.221
	(0.094)	(0.503)	(0.281)
pgdp	−0.027	0.147	0.081
	(0.022)	(0.235)	(0.077)
aging	−0.592^***^	−1.966	1.572^***^
	(0.226)	(1.404)	(0.512)
den	−0.004	0.080	−0.041^**^
	(0.009)	(0.065)	(0.020)
edu	−0.133	−1.598^*^	−0.099
	(0.109)	(0.864)	(0.180)
hicr	0.162	3.132^***^	0.053
	(0.099)	(1.072)	(0.205)
Constant	0.340	−1.289	0.282
	(0.240)	(2.218)	(0.656)
AR (1)	0.011	0.012	0.002
AR (2)	0.129	0.163	0.323
Sargan	0.674	1.000	1.000
Sample size	279	279	279

**Table 9:** Results of the mechanism test for MAS_M (measured by coefficient of variation)

Variables	(1)	(2)	(3)
	MAS_M	OUE	IUE
L.OUE		0.865^***^	
		(0.096)	
L.IUE			0.737^***^
			(0.034)
L.MAS_M	1.015^***^		
	(0.093)		
HG	0.601^***^	−4.806^***^	−1.023^*^
	(0.212)	(1.785)	(0.552)
MAS_M		−2.521^**^	−0.308^*^
		(1.219)	(0.169)
fhexp	−0.024	−2.358	1.409^*^
	(0.351)	(3.102)	(0.816)
urban	−0.009	−2.171	−0.215
	(0.065)	(1.821)	(0.250)
pgdp	−0.048	1.060^*^	0.099
	(0.032)	(0.630)	(0.090)
aging	0.130	−1.829	1.679^***^
	(0.195)	(2.779)	(0.557)
den	−0.018	−0.035	−0.041
	(0.019)	(0.104)	(0.026)
edu	−0.006	−1.853^*^	−0.098
	(0.054)	(0.965)	(0.168)
hicr	−0.096	4.140^***^	0.151
	(0.079)	(1.195)	(0.209)
Constant	0.650	−7.316^*^	0.146
	(0.424)	(4.417)	(0.731)
AR (1)	0.021	0.010	0.002
AR (2)	0.141	0.505	0.287
Sargan	0.997	0.994	1.000
Sample size	279	279	279

Consistent with the analysis in Table 7, the regression results in Tables 8 and 9 indicate that the hospital grading system exacerbates the disparities in the allocation of both medical human and material resources between hospitals and primary healthcare institutions, thereby leading to lower levels of both outpatient and inpatient resource utilization. Furthermore, these results also demonstrate that the mechanism tests in this study are robust.

When using the Theil index to measure the medical resource allocation structure (TMAS), the regression results of the mechanism test are presented in Table 10. Column (1) of Table 10 presents the estimation results with the overall weighted medical resource allocation structure (TMAS) as the explained variable, demonstrating that the conclusion that the hospital grading system exacerbates disparities in medical resource allocation remains valid even after changing the measurement method of the indicator. In Columns (2) and (3) of Table 10, the coefficient of the core explanatory variable, HG, is significantly negative. Comparing these results with the baseline regression in Table 3, the negative effect of HG weakens, with its coefficient changing from −5.675 to −5.481 and from −2.029 to −1.089, respectively. The findings suggest that even after altering the measurement method, the hospital grading system continues to have a significant impact on the disparities in medical resource allocation. This further affirms the robustness of the mechanism test results.

**Table 10:** Results of the mechanism test for TMAS (measured by Theil Index)

Variables	(1)	(2)	(3)
	TMAS	OUE	IUE
L.OUE		0.859^***^	
		(0.082)	
L. TMAS	0.939^***^		
	(0.051)		
L.IUE			0.740^***^
			(0.033)
HG	0.096^***^	-5.481^**^	-1.089^*^
	(0.033)	(2.487)	(0.607)
TMAS		-7.995^*^	-1.056^*^
		(4.642)	(0.617)
fhexp	0.043	-3.141	1.316
	(0.049)	(2.873)	(0.871)
urban	0.020^*^	-0.399	-0.252
	(0.011)	(0.975)	(0.259)
pgdp	-0.006^***^	0.406	0.081
	(0.002)	(0.327)	(0.083)
aging	-0.043^*^	0.432	1.714^***^
	(0.023)	(1.832)	(0.556)
den	-0.003	0.003	-0.042^*^
	(0.002)	(0.097)	(0.023)
edu	-0.014	-1.395	-0.065
	(0.010)	(1.191)	(0.174)
hicr	-0.016	4.821^***^	0.162
	(0.016)	(1.117)	(0.218)
Constant	0.084^***^	-2.953	0.248
	(0.032)	(2.946)	(0.680)
AR (1)	0.001	0.019	0.002
AR (2)	0.183	0.510	0.307
Sargan	0.999	0.990	1.000
Sample size	279	279	279

Overall, the findings suggest that optimizing the structure of medical resource allocation is essential for improving the performance of the healthcare system. Key areas to prioritize in China’s healthcare system reform include refining the hospital grading system to ensure a more equitable distribution of resources across healthcare institutions, increasing investment and resource allocation in primary healthcare institutions, and promoting partnerships and resource-sharing mechanisms between high-grade hospitals and primary healthcare institutions. In contrast to previous studies, this study not only provides quantitative evidence of the hospital grading system’s association with lower medical resource utilization but also clarifies the specific mechanisms through which this relationship occurs, offering valuable insights for policymakers.

## Conclusions and discussion

### Conclusions

This study systematically analyzes the relationships and mechanisms of the hospital grading system and medical resource utilization. The main conclusions are as follows:

(1) The hospital grading system shows a significant negative relationship with the level of medical resource utilization. Subgroup analyses suggest broadly consistent negative effects on outpatient utilization across both eastern and central–western regions, with the negative effect being substantially larger in magnitude for the eastern region. For inpatient utilization, significance is only observed in the central-western region. Analysis by resource endowment shows that outpatient utilization is significantly impaired in high-resource regions, while inpatient utilization is significantly lower in low-resource regions. The differential magnitudes across subgroups suggest potential heterogeneity that warrants further formal testing. (2) In recent years, there has been an increasing disparity in medical human resource allocation structure between hospitals and primary healthcare institutions in China. Specifically, the disparity in the allocation of registered nurses is more pronounced than that of practicing (assistant) physicians. Regarding medical material resources, the disparity in bed and equipment capacity between hospitals and primary healthcare institutions has further widened. (3) Mechanism analysis suggests that the hospital grading system is indirectly associated with a reduction in medical resource utilization, primarily through exacerbating the medical resource allocation gap between hospitals and primary healthcare institutions.

### Policy implications

The empirical findings of this study offer several policy implications for reforming the current hospital grading system, optimizing the medical resource allocation structure, and enhancing the effectiveness of medical resource utilization. While these insights are particularly relevant for China, they may also offer valuable lessons for countries with similar healthcare systems and socio-economic conditions. The policy implications of this study can be summarized in three main aspects.

First, the grading criteria should be reformed by shifting from hardware-based metrics to outcome-based indicators. This study suggests that the current hospital grading system is associated with lower medical resource utilization, as it incentivizes hospitals to compete on scale (e.g., beds, equipment) rather than actual service quality and effectiveness. Therefore, it is recommended that the weight of quantitative hardware indicators be reduced and reallocated to metrics focused on resource utilization and patient outcomes, such as patient satisfaction, referral rates, and the quality of technical support provided to lower-tier institutions. Additionally, a third-party, market-oriented evaluation body should be introduced to ensure objectivity and reduce reliance on purely administrative assessments.

Second, policy interventions should focus on narrowing the material resource gap for primary healthcare institutions. The mechanism analysis in this study suggests that the grading system is associated with lower medical resource utilization by widening the disparity in material resources between hospitals and primary institutions. Crucially, the findings indicate that the disparity in material resources (e.g., beds, medical equipment) is more pronounced than that in human resources. Accordingly, financial and policy support should prioritize upgrading medical equipment and expanding facility capacity at primary healthcare institutions. Simultaneously, personnel management reforms are needed to mitigate the brain drain of healthcare professionals from primary institutions, particularly through incentive schemes that make primary care practice more attractive.

Third, policy responses should be tailored to address regional heterogeneity identified in this study. The results reveal distinct patterns of inefficient resource utilization across regions with varying resource endowments. Therefore, region-specific approaches are essential. In high-resource regions (where outpatient utilization is more strongly impacted), reforms should emphasize optimizing outpatient services and enhancing coordination between hospitals and primary healthcare institutions. In low-resource regions (where inpatient utilization is most affected), efforts should focus on improving inpatient care efficiency through additional investments in primary healthcare infrastructure and a more balanced distribution of medical personnel. Particular attention should be devoted to central-western regions, where this study identifies especially pronounced severe negative impacts on inpatient utilization.

Finally, these reforms involve inevitable trade-offs, such as potential resistance from large hospitals and fiscal pressures, which call for gradual and coordinated implementation. Nonetheless, the challenges of resource concentration and weak primary care observed in China are present across many developing countries. Therefore, the findings and recommendations of this study should be viewed as potential references for similar contexts.

### Limitations and future research

Although this study provides valuable insights into the relationship between the hospital grading system and medical resource utilization, it has certain limitations.

First, the analysis primarily relies on panel data from 31 provinces in mainland China spanning from 2010 to 2019, due to its availability. In particular, the study uses the proportion of tertiary hospitals as a proxy for the implementation of the hospital grading system. While this indicator reflects structural changes in the healthcare system, it cannot fully capture other important dimensions of the policy, such as referral mechanisms, inter-level coordination, or enforcement. Although provincial-level panel data allow this study to capture cross-provincial differences in healthcare delivery and resource utilization, they inevitably aggregate conditions within each province and may obscure substantial intra-provincial heterogeneity. Therefore, future research should focus on collecting more detailed data at the micro-level within healthcare institutions and incorporate richer data sources to examine these aspects more comprehensively.

Second, this study explores the pathways through which the hospital grading system is associated with lower medical resource utilization, focusing on the role of the medical resource allocation structure. While this provides a useful starting point, future research could further explore the impact of the hospital grading system by separately analyzing its effects on medical resource utilization in high-grade healthcare institutions and primary healthcare institutions. This approach could help identify unique challenges and opportunities within each type of institution, thereby enhancing the hospital grading system’s effectiveness in a more targeted manner.

Third, while this study primarily focuses on the hospital grading system and medical resource utilization across Chinese provinces, future research could broaden its scope to include international studies across various countries. This could deepen the understanding of how similar hospital grading systems influence medical resource utilization, offering valuable cross-national insights for healthcare policy improvements worldwide. In addition, unmeasured confounders—such as regional differences in policy enforcement, variations in health-seeking behavior, and disparities in institutional capacity—may also influence the observed outcomes. Future work should incorporate these dimensions to provide a more robust and comprehensive analysis.

## Data Availability

Data is available from the corresponding author on request.

## Footnotes

1 This study confirmed the feasibility of this supplementary method by cross-verifying it with data from additional published years.
